# Computing Average Passive Forces in Sarcomeres in Length-Ramp Simulations

**DOI:** 10.1371/journal.pcbi.1004904

**Published:** 2016-06-08

**Authors:** Gudrun Schappacher-Tilp, Timothy Leonard, Gertrud Desch, Walter Herzog

**Affiliations:** 1 Department for Mathematics and Computational Sciences, University of Graz, Graz, Austria; 2 Human Performance Laboratory, University of Calgary, Calgary, Canada; University of California San Diego, UNITED STATES

## Abstract

Passive forces in sarcomeres are mainly related to the giant protein titin. Titin’s extensible region consists of spring-like elements acting in series. In skeletal muscles these elements are the PEVK segment, two distinct immunoglobulin (Ig) domain regions (proximal and distal), and a N2A portion. While distal Ig domains are thought to form inextensible end filaments in intact sarcomeres, proximal Ig domains unfold in a force- and time-dependent manner. In length-ramp experiments of single titin strands, sequential unfolding of Ig domains leads to a typical saw-tooth pattern in force-elongation curves which can be simulated by Monte Carlo simulations. In sarcomeres, where more than a thousand titin strands are arranged in parallel, numerous Monte Carlo simulations are required to estimate the resultant force of all titin filaments based on the non-uniform titin elongations. To simplify calculations, the stochastic model of passive forces is often replaced by linear or non-linear deterministic and phenomenological functions. However, new theories of muscle contraction are based on the hypothesized binding of titin to the actin filament upon activation, and thereby on a prominent role of the structural properties of titin. Therefore, these theories necessitate a detailed analysis of titin forces in length-ramp experiments. In our study we present a simple and efficient alternative to Monte Carlo simulations. Based on a structural titin model, we calculate the exact probability distributions of unfolded Ig domains under length-ramp conditions needed for rigorous analysis of expected forces, distribution of unfolding forces, etc. Due to the generality of our model, the approach is applicable to a wide range of stochastic protein unfolding problems.

## Author Summary

We provide a simple and stable algorithm to determine the exact solution of passive forces in a half sarcomere in length-ramp simulations. The approach is applicable to a wide range of stochastic models of protein unfolding.

## Introduction

**Fig 1:**
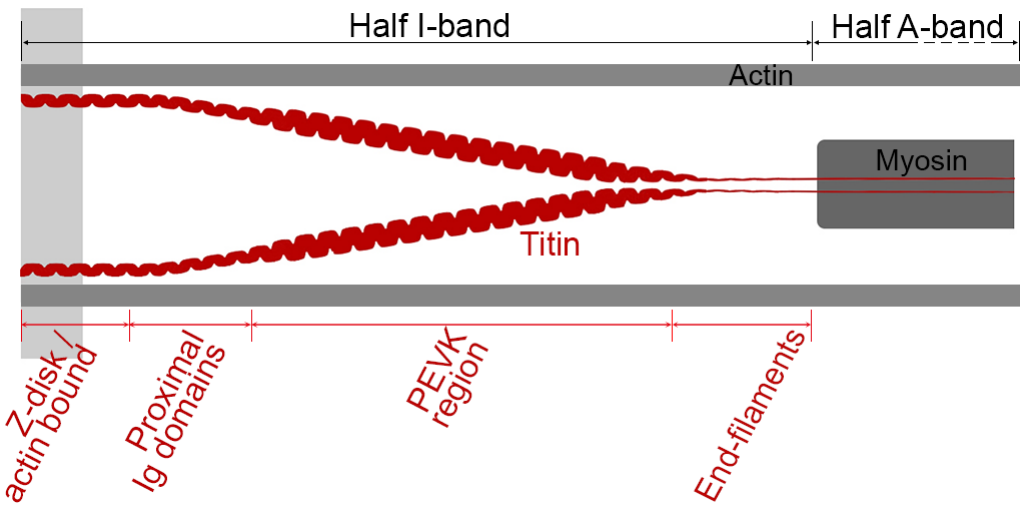
Sketch of titin strands in a half sarcomere. While a single titin strand spans the whole half sarcomere only a part of titin located in the half I-band is extensible.

Passive forces in sarcomeres or myofibrils are almost exclusively governed by the giant protein titin [1]. A titin strand spans the half sarcomere from Z-disk to M-band. While its section located in the thick filament is nearly inextensible, its I-band region functions as a molecular spring. In skeletal muscles, titin’s I-band region comprises two immunoglobulin (Ig) domains, a N2A portion as well as a region rich in proline(P), glutamate (E), valine (V), and lysine(K), the PEVK region [1, 2]. The distal Ig domains (close to the AI-junction) are thought to form almost inextensible end-filaments [3, 4] (Fig 1).

The proximal Ig domains (close to the Z-disk) are able to unfold in a force and time dependent manner [5]. Single molecule atomic force microscopy, in which the length of a single molecule is controlled, show a characteristic sawtooth pattern in force-extension curves due to a complex time series of unfolding events, e.g. [6–10]. Molecular dynamic simulations coincide with experimental data and provide new insight into the mechanics of unfolding at the atomic level; e.g. [11–14].

In a half sarcomere around 1.2⋅10^9^ titin strands per mm² are arranged in parallel [15, 16]. Therefore, the sawtooth pattern in half-sarcomers is completely averaged out. However, it is still possible to observe unfolding events in myofibrils implicitly because of a prominent change of stiffness [17, 18].

A favourable experimental set-up to study unfolding characteristics, like mean dwell times or the distribution of forces at rupture of single proteins, are so-called force-clamp studies and force-ramp studies. In force-clamp studies the force is maintained at a constant level whereas in force-ramp studies the force increases linearly [19]. Elongation of protein length due to unfolding events is then a step function over time [20, 21]. Experimental data can be rigorously analysed by order statistics [22, 23].

However, the theoretical framework of force-clamp analysis is not applicable in length-ramp experiments which are frequently used in myofibril studies, like [24–28] to name a few. Models of active and passive force production under dynamic conditions on the sarcomere or myofibril level either use Monte Carlo simulations of single titin strands where single protein forces are scaled to half sarcomere forces, e.g. [29], or phenomenological models, e.g. [30, 31]. Monte Carlo simulations of single titin strands deliver detailed information about the possible behaviour of single titin strands which is not necessarily needed for models on the half sarcomere level. On the other hand the characteristics of force production on the sarcomere level are still only an approximation. Phenomenological models focus on the characteristics of passive forces in half sarcomeres thereby condensing the information but they might not be sufficient when dealing with new theories on muscle contraction involving titin binding to actin upon activation (e.g. [25, 29, 32, 33]). These theories require knowledge of titin’s mechanical behavior as well as the mechanics of isolated titin segments

In this work we are interested in the forces contributed by all titin strands in a half sarcomere. Since titin strands are aligned in parallel the total force is the sum of single protein forces and thereby the weighted expectation value of a single molecule. We reformulate a well known titin model which is based on the kinetic properties of titin’s macroscopic structures and therefore easily adjustable to various theories of muscle contraction. We define a simple two state unfolding regime and derive the exact probabilities describing the regime. Finally, we compare our results to Monte Carlo approximations and previously published results, as well as our own experimental data. Our approach provides a simple algorithm to calculate the exact expectation value of forces contributed by titin in a half sarcomere or probability distribution of unfolding forces.

## Models

Throughout the manuscript we base our simulations on rabbit psoas 3.400-kD isoform [34], i.e. 50 proximal Ig domains, 800 PEVK residues and 26 distal Ig domains. However, the isoform only affects numerical results but not the theoretical considerations.

The stochastic model in a slightly different form, and the corresponding Monte Carlo simulations have been published before, e.g. [7, 29, 35–37]. In this work, we provide a rigorous but simple mathematical formulation of the model allowing for a straightforward deduction of the existence of a unique solution of the stochastic model. In addition, we include the possible hierarchy of unfolding [36] or (mathematically equivalent) different mechanical stability of Ig domains [38] in both, Monte Carlo approximations and the exact solution.

To allow an effective description of the theoretical considerations, we formulate the theoretical framework for stretching experiments where refolding is highly unlikely. Finally, we introduce refolding events for a simplified case.

### Titin model based on the kinetics of its microscopic structures

Single-molecule experiments suggest that titin’s Ig domains (folded or unfolded) act like a molecular spring showing wormlike chain (WLC) behaviour [9]. Upon unfolding the extended Ig domain gains the distance *d*_*u*_. Titin’s PEVK region shows modified WLC behavior [16]. A well known approximation to the WLC model of entropic elasticity [39, 40] relates the external force *F* to the end to end length *x* of the chain by

$$\begin{array}{c} F=\frac{k_{B}T}{pl}\left(\frac{1}{4{\left(1-\frac{x}{cl}\right)}^{2}}-\frac{1}{4}+\frac{x}{cl}\right), \end{array}$$ (1)

where *pl* is the persistence length and *cl* the chain’s contour length, *k*_*B*_ is the Boltzmann constant and *T* the absolute temperature. The modified WLC model includes an enthalpic contribution to elasticity by including a stretch modulus *F*_0_:

$$\begin{array}{c} F=\frac{k_{B}T}{pl}\left(\frac{1}{4{\left(1-\frac{x}{cl}+\frac{F}{F_{0}}\right)}^{2}}-\frac{1}{4}+\frac{x}{cl}-\frac{F}{F_{0}}\right). \end{array}$$ (2)

The stochastic model describing the force contributed by a single titin strand in a half sarcomere with length *l* can be expressed as a convex optimization problem:

$$\begin{array}{c} \begin{array}{c} \underset{l_{P\;E\;V\;K},l_{f},l_{u},l_{e\;n\;d},l_{r\;e\;s\;t}}{\text{minimize}}\left(V_{f}\left(l_{f}\right)+V_{u}\left(l_{u}\right)+V_{P\;E\;V\;K}\left(l_{P\;E\;V\;K}\right)+V_{end}\left(l_{e\;n\;d}\right)+V_{r\;e\;s\;t}\left(l_{r\;e\;s\;t}\right)\right)\\ \text{subject to}\left\{\begin{array}{c} l=l_{P\;E\;V\;K}+l_{f}n_{f}+l_{u}\left(N-n_{f}\right)+l_{e\;n\;d}+l_{r\;e\;s\;t}\\ l_{P\;E\;V\;K},l_{f},l_{u},l_{e\;n\;d},l_{r\;e\;s\;t}\geq 0 \end{array}\right., \end{array} \end{array}$$ (3)

where *l*_*PEVK*_, *l*_*f*_, *l*_*u*_, *l*_*end*_ and *l*_*rest*_ are the lengths of PEVK, folded and unfolded Ig domains, end-filaments as well as the sum of titin’s A-band region and slack length respectively. *V*^*u*, *f*^ is the potential of the WLC model for unfolded and folded proximal Ig domains, *V*_*PEVK*_ is the potential of the modified WLC model. *V*_*end*_ and *V*_*rest*_ are potentials of very stiff linear springs describing the almost in-extensible end-filaments formed by distal Ig domains, and titin’s A-band region and slack length, respectively. *N* is the total number of Ig domains. The number of folded Ig domains *n*_*f*_ is a discrete random variable. The formulation of the optimization problem guarantees the existence of a unique solution of Eq (3) whenever the number of folded Ig domains is known and the convex feasible set is non-empty.

Mechanical model parameters are average values obtained from the literature and provided in Table 1.

**Table 1:** Mechanical model parameters [16, 36, 41, 42].

*pl*_*PEVK*_	6⋅10^−10^m	*k*_*bound*_	2Nm^−1^
*cl*_*PEVK*_	3.6⋅10^−10^m	*k*_*rest*_	2Nm^−1^
*F*_0_	150⋅10^−12^N	*x*_*u*_	0.25nm
*pl*_*Ig*_	8.5⋅10^−10^m	*x*_*f*_	2.2nm
*cl*_*Ig*_	4.5⋅10^−10^m	*d*_*u*_	25nm

**Fig 2:**
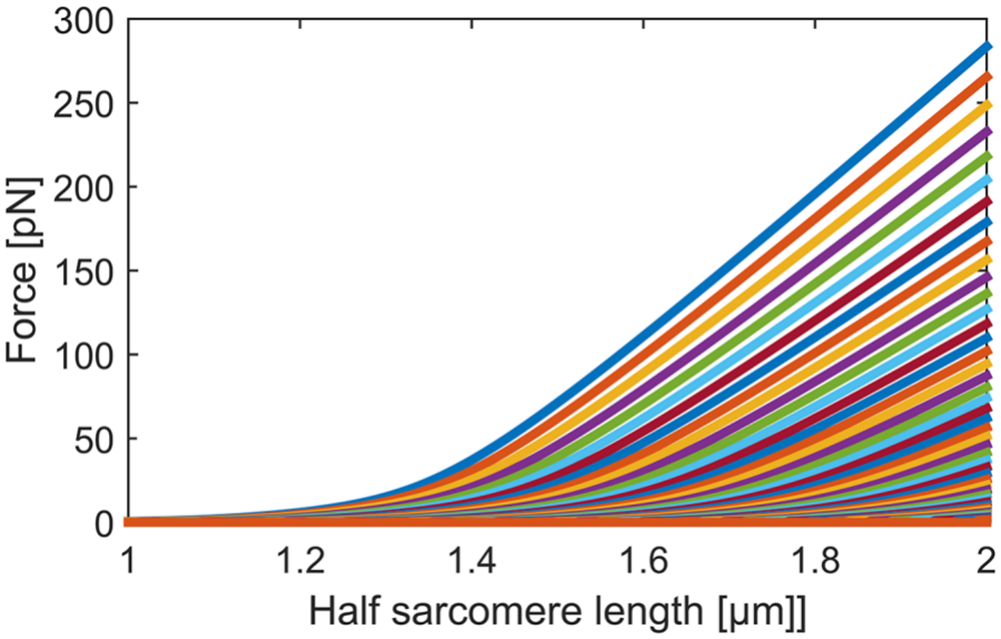
Solution of Eq (3) for stretches from 1μm to 2μm half sarcomere lengths. Force [pN] of single titin strands at a given half sarcomere length is a discrete random variable with 51 states. The top curve represents the state in which all Ig domains are folded, the next curve the state in which exactly one Ig domain is unfolded, etc.

Since we are interested in simulating length-ramp experiments, we solve Eq (3) for every possible *n*_*f*_ (see Fig 2) for half sarcomere lengths starting at 1μm. With *F*_*i*_(*l*(*t*)) = *F*_*i*_(*t*), *i* = 0, …, 50 we denote the force as a function of length (and hence time) when exactly *i* Ig domains are unfolded.

Unfolding and refolding of one Ig domain is a thermally driven process described by the unfolding rate function *u* and the folding rate function *f*. The barrier height of the unfolding and refolding process is influenced by the external force *F*:

$$\begin{array}{c} u\left(F\right)=\omega_{0}e^{\frac{Fx_{u}}{k_{b}T}},r\left(F\right)=\omega_{1}e^{-\frac{Fx_{f}}{k_{b}T}}, \end{array}$$ (4)

where *ω*_0_ and *ω*_1_ are spontaneous unfolding / folding rates at zero force and *x*_*u*_, *x*_*f*_ are the width of the activation barriers, e.g. [35]. Due to the length-ramp condition the force changes significantly with every unfolding event thereby directly influencing the unfolding probability of the remaining folded Ig domains.

Since there is experimental support for hierarchical unfolding of Ig domains [36], or differences in the mechanical stability of Ig domains homogeneously distributed across the titin strands [38], we introduce *m* Ig clusters, where each cluster has a different spontaneous unfolding rate *ω*_0_. We denote the different unfolding rates by *u*_*k*_, *k* = 1, …, *m* and assume (without loss of generality) that *u*_1_(*F*) ≤ *u*_2_(*F*) ≤ … ≤ *u*_*m*_(*F*) for any given force *F*. It is worth pointing out that we could define the clusters by different activation barriers if it would prove favourable in terms of parameter estimation or data fitting. However, such an approach would not change the characteristics of the simulations presented in this paper.

### Monte Carlo simulations

We simulate *R* titin strands and approximate the expected forces in a half sarcomere by averaging the forces of *R* titin strands and scaling the force by the number of titin strands in a half sarcomere. For a single titin strand let *m* be the number of unfolding clusters. We simulate stretches starting at low half sarcomere lengths where unfolding events are highly unlikely. Therefore, we start with 50 folded Ig domains. The probability *p*_*k*_ that one Ig domain of the *k*-th cluster with *n*_*k*_ Ig domains will unfold within the next time step is approximately *p*_*k*_(*l*(*t*_1_)) = *n*_*k*_ ⋅ *u*_*k*_(*F*_0_(*l*(*t*_1_))) ⋅ *dt*. By drawing an equally distributed random number 0 ≤ *z* ≤ 1, we define an unfolding event in the *k*-th cluster if

$$\begin{array}{c} \begin{array}{ccc} \sum_{j=1}^{k-1}p_{j}\left(l\left(t_{1}\right)\right) & <z\leq & p_{k}\left(l\left(t_{1}\right)\right)\\ \text{or }\sum_{j=1}^{k-1}n_{j}\cdot u_{j}\left(F_{0}\left(t_{1}\right)\right)\cdot dt & <z\leq & n_{k}\cdot u_{k}\left(F_{0}\left(t_{1}\right)\right)\cdot dt \end{array} \end{array}$$ (5)

If no cluster fulfils condition (5), no unfolding event takes place. In a similar way, we determine an unfolding event at any other time step *t*_*i*_, where *k*_1_, *k*_2_, …, *k*_*m*_ Ig proteins of the first, second,…,*m*th cluster are already unfolded, respectively. Again, let 0 ≤ *z* ≤ 1 be an equally distributed random number. Let $N_{f}=N-\sum_{j=1}^{m}k_{j}$. We define an unfolding event in the *l*-th cluster if

$$\begin{array}{c} \sum_{j=1}^{l-1}\left(n_{j}-k_{j}\right)\cdot u_{j}\left(F_{N_{f}}\left(t_{i}\right)\right)\cdot dt<z\leq \left(n_{l}-k_{l}\right)\cdot u_{l}\left(F_{N_{f}}\left(t_{i}\right)\right)\cdot dt \end{array}$$ (6)

If refolding has to be taken into account, e.g., simulation of hysteresis curves [5, 17, 18], the formula can be adjusted accordingly. For the sake of simplicity, we introduce the formula for one cluster which can be easily generalized to an arbitrary amount of clusters: Let *i* Ig domains be unfolded at some time *t*. The probability that a folded Ig domain will unfold within the next time step *t* + *dt* is approximately (*n* − *i*) ⋅ *u*(*F*_*i*_(*l*(*t* + *dt*))) ⋅ *dt*, while the probability that one of the *i* unfolded Ig domains will refold within the next time step is approximately *i* ⋅ *r*(*F*_*i*_(*l*(*t* + *dt*))) ⋅ *dt*. By drawing an equally distributed random number 0 ≤ *z* ≤ 1, we define an unfolding event if

$$\begin{array}{c} z\leq \left(n-i\right)\cdot u(F_{i}\left(l\left(t+dt\right)\right))\cdot dt, \end{array}$$ (7)

and a refolding event if

$$\begin{array}{c} \left(n-i\right)\cdot u\left(F_{i}\left(l\left(t+dt\right)\right)\right)\cdot dt<z\leq \left(n-i\right)\cdot u\left(F_{i}\left(l\left(t+dt\right)\right)\right)\cdot dt+i\cdot r\left(F_{i}\left(l\left(t+dt\right)\right)\right)\cdot dt. \end{array}$$ (8)

Finally, we determined the number *R* of simulated titin strands by estimating the error of Monte Carlo simulations. Specifically, we performed *R* independent Monte Carlo simulations *H* times to estimate the mean force *F*_*h*_(*l*_*i*_) at a given length *l*_*i*_, *i* = 1, …, *L* for *h* = 1, …, *H*. We define the (*R* dependent) error of the Monte Carlo simulation *MCE* by

$$\begin{array}{c} M\;C\;E{\left(R\right)}^{2}={\left\|\langle F_{h}^{2}\left(\cdot \right)\rangle -{\langle F_{h}\left(\cdot \right)\rangle }^{2}\right\|}_{\infty }, \end{array}$$

where $\langle x\rangle =\left(1/N\right)\cdot \sum_{n=1}^{N}x_{i}$ for *x* = (*x*_1_, …, *x*_*n*_). For *H* = 100 we get the following *MCE* estimates: *MCE*(10) = 2.71, *MCE*(50) = 1.3, *MCE*(100) = 0.84, *MCE*(200) = 0.63, *MCE*(500) = 0.39. Therefore, seeking a compromise between simulation time and accuracy, we chose to simulate 200 titin strands.

### Exact solution

For the exact solution, we need to calculate the probabilities that after a stretch to a given length *l*(*t*) no unfolding event took place (*P*_0_), exactly one unfolding event took place (*P*_1_), up to *N* unfolding events took place (*P*_*N*_). The mathematical formulation of the probabilities is straight forward. For instance, *P*_0_ is the survival probability of all Ig domains, i.e.

$$\begin{array}{c} P_{0}\left(t\right)=\exp\left(-\sum_{k=1}^{m}n_{k}\int_{0}^{t}u_{k}\left(F_{0}\left(\tau \right)\right)d\tau \right). \end{array}$$ (9)

*P*_1_(*t*) is the probability, that no unfolding takes place until time *τ*, then one Ig domain of one of the *m* clusters unfolds, and then no further event takes place between *τ* and *t*, i.e.

$$\begin{array}{c} P_{1}\left(t\right)=\sum_{l=1}^{m}\int_{0}^{t}P_{0}\left(\tau \right)u_{k}\left(F_{0}\left(\tau \right)\right)\exp\left(-\sum_{i=1}^{m}\left(n_{i}-\delta_{i,l}\right)\int_{\tau }^{t}u_{i}\left(F_{1}\left(\sigma \right)\right)d\sigma \right)d\tau , \end{array}$$ (10)

where $\delta_{ij}=\{\begin{array}{cc} 0, & i\neq j\\ 1, & i=j \end{array}$.

However, due to the non-linear unfolding rate function and forces we cannot solve Eqs (9) and (10) analytically and the numerical solution is unstable. Therefore, it is already highly costly to compute *P*_1_, and costs are rising with every unfolded Ig domain. We therefore define basic probabilities which add up to the desired ones. Let *p*_*i*_1_*i*_2_….*i*_*m*__(*t*) be the probability that at a given time *t*, *i*_1_ proteins of the first cluster, *i*_2_ of the second, …., and *i*_*m*_ of the *m*-th cluster are unfolded. The dynamics of these probabilities can be described with a simple system of linear ordinary differential Eq (11).

$$\begin{array}{c} \begin{array}{ccc} dp_{i_{1}....i_{m}}/dt & = & -\left(\sum_{l=1}^{m}\left(n_{l}-i_{l}\right)\cdot u_{l}\left(F_{i_{1}+...+i_{m}}\left(t\right)\right)\right)p_{i_{1}....i_{m}}\left(1-\delta_{i_{l},n_{l}}\right)\\ & & +\left(n_{1}-i_{1}+1\right)u_{1}\left(F_{i_{1}+...+i_{m}-1}\left(t\right)\right)p_{\left(i_{1}\;-\;1\right)i_{2}...i_{m}}\left(1-\delta_{i_{1},0}\right)+...\\ & & +\left(n_{m}-i_{m}+1\right)u_{1}\left(F_{i_{1}+...+i_{m}-1}\left(t\right)\right)p_{i_{1}i_{2}...\left(i_{m}\;-\;1\right)}\left(t\right)\left(1-\delta_{i_{m},0}\right),\\ & & 0\leq i_{1}\leq n_{1},...,0\leq i_{m}\leq n_{m} \end{array} \end{array}$$ (11)

This detour has the advantage that we can solve the system fast and stable by an implicit Euler method. Finally, we calculate

$$\begin{array}{c} P_{l}=\sum_{\lambda \in \Lambda_{l}}p_{\lambda },\;\;l=0,...,N, \end{array}$$ (12)

where $\Lambda_{l}=\left\{\left(i_{1},...,i_{m}\right),0\leq i_{1}\leq n_{1},...,0\leq i_{m}\leq n_{m},\sum_{k=1}^{m}i_{k}=l\right\}$. The expected force in a half sarcomere is then

$$\begin{array}{c} E\left(F\left(t\right)\right)=\sum_{l=0}^{N}P_{l}\left(t\right)F_{l}\left(t\right). \end{array}$$ (13)

Again, if refolding has to be taken into account the adjustment of the formulae is straight forward. For example, the formulation of refolding and folding of one cluster reads

$$\begin{array}{c} \begin{array}{ccc} dp_{i}/dt & = & \left(n-i+1\right)u\left(F_{i-1}\left(t\right)\right)p_{i-1}\left(t\right)+\left(n-i-1\right)r\left(F_{i+1}\left(t\right)\right)p_{i+1}\left(t\right)\\ & & -\left(n-i\right)u\left(F_{i}\left(t\right)\right)p_{i}\left(t\right)-ir\left(F_{i}\left(t\right)\right)p_{i}\left(t\right), \end{array} \end{array}$$ (14)

where *p*_*i*_(*t*) is the probability that *i* Ig domains are folded at time *t* and *r* is the refolding rate.

### Experiments using single myofibrils

Briefly, single myofibrils isolated from rabbit psoas muscle were wrapped around a glass needle at one end and fixed to a nanolever at the other end. The experimental set-up allowed for length change and force measurements, respectively. Myofibrils were passively stretched in a non-activating (pCa 8.0) solution containing ATP. The experimental procedure is described in detail elsewhere [25, 43]. Since extracellular connective tissues are absent in a single myofibril, all forces are attributed to proteins comprising sarcomeres in series. Due to sarcomere non-uniformities the scaling from a half sarcomere to a myofibril is not trivial. However, properties like hysteresis or the smoothing of unfolding events in a half sarcomere are reflected in single myofibrils [18]. Ethics approval for the single myofibril experiments was granted by the Life and Environmental Sciences Animal Ethics Committee of the University of Calgary.

## Results

**Fig 3:**
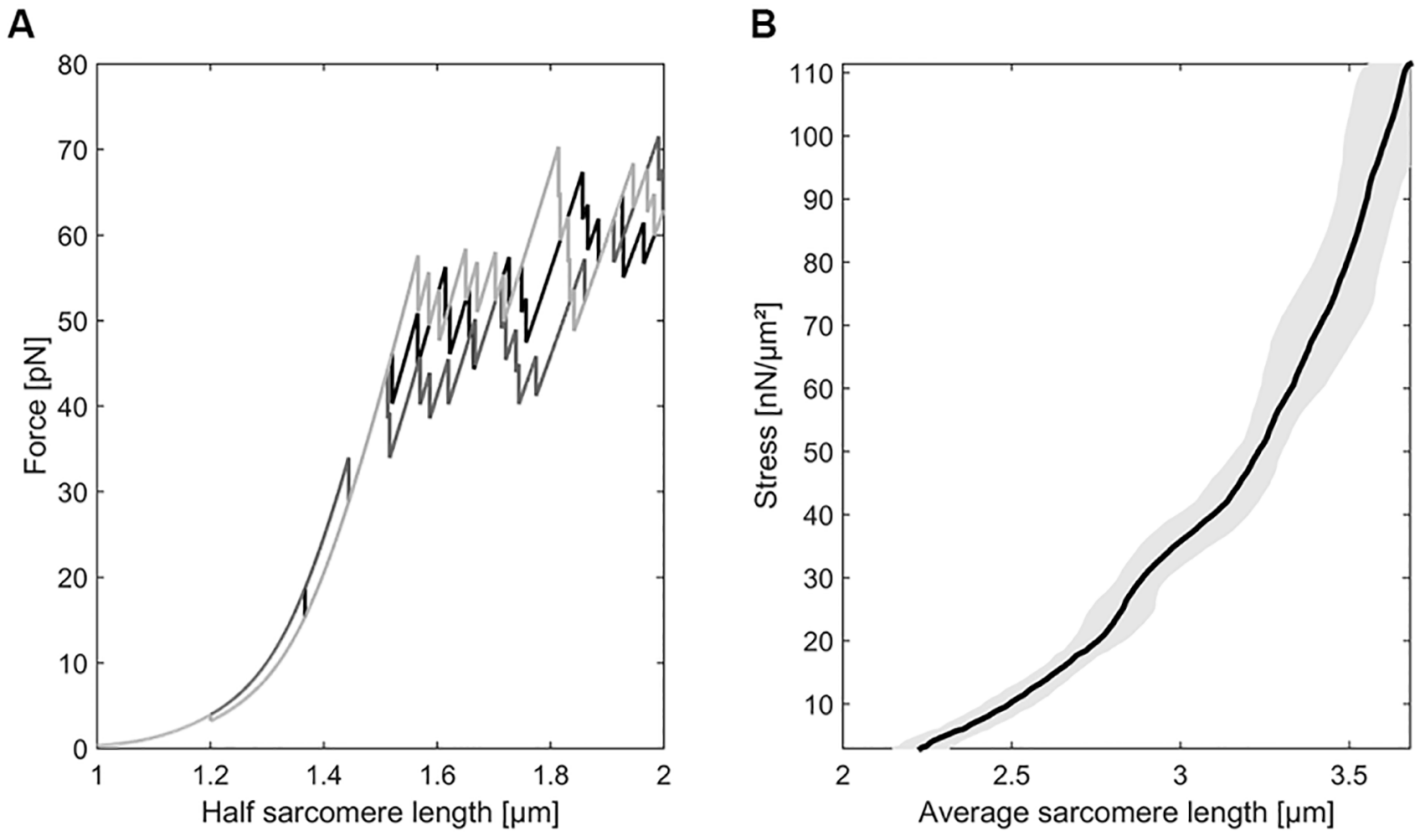
Force-elongation behavior of a single titin strand with corresponding Monte Carlo simulations and force-elongation relation of a myofibril. **A** When a single titin molecule is stretched it shows a typical saw-tooth pattern due to the unfolding of Ig domains [5, 9, 36, 38]. Three exemplar Monte Carlo simulations simulate that behavior. **B** Force-elongation curves of two myofibrils consisting of 7 and 8 sarcomeres respectivley. The shaded region indicates the standard error of the mean sarcomere length. Since over 1000 titin molecules are arranged in parallel in a single half sarcomere the saw tooth pattern observed in single molecule experiments is averaged out.

The first simulations are based on 5 different unfolding clusters, each cluster covering 10 proximal Ig domains. Monte Carlo simulations of single titin strands show the typical saw-tooth pattern [5, 9, 36, 38] while this pattern is completely averaged out in a short myofibril comprised of 7 sarcomere (Fig 3).

**Fig 4:**
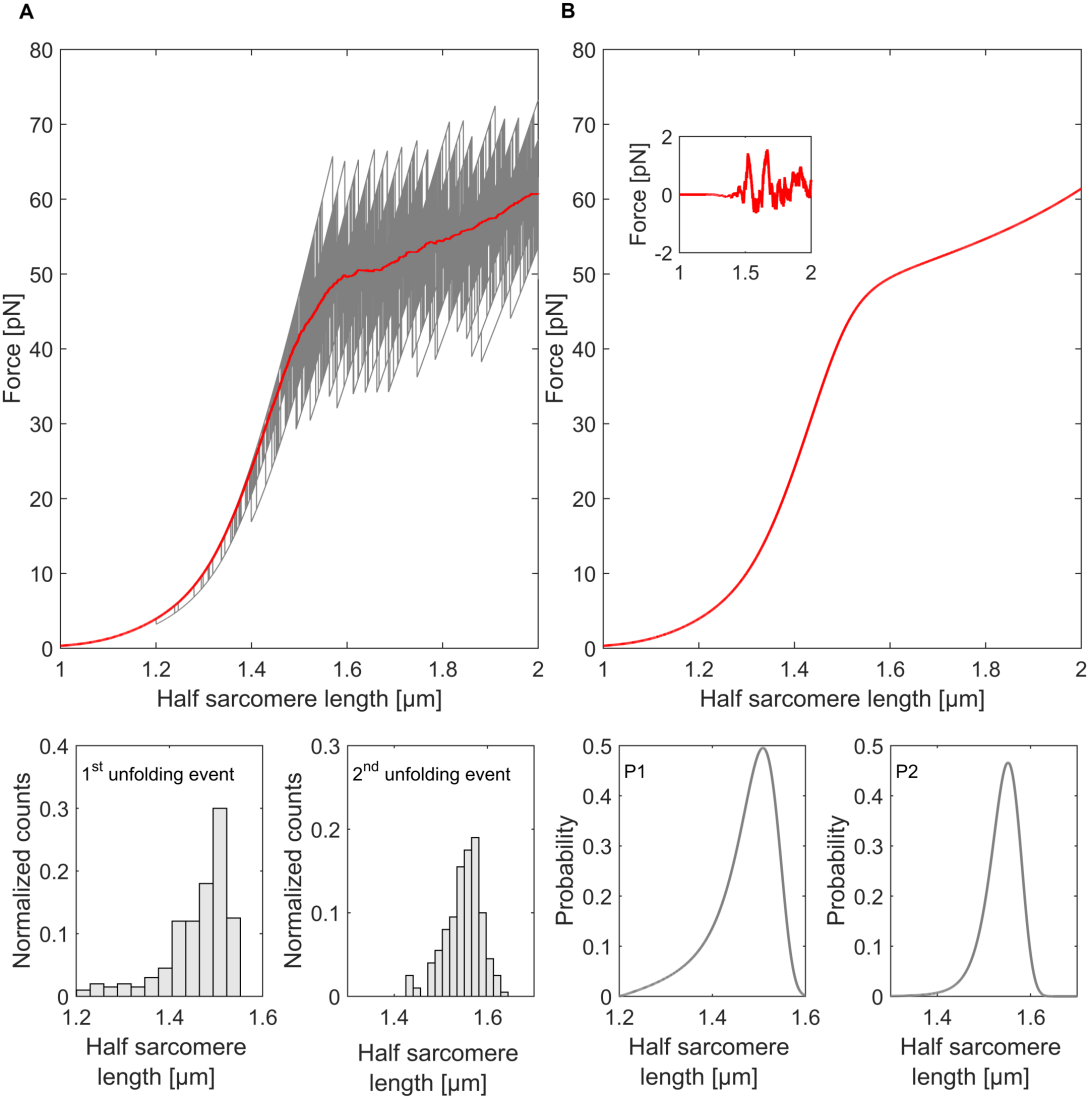
Comparison between the two methods. **A** Force-elongation curves of the Monte Carlo simulation. The red line indicates the mean value of force at a given half sarcomere length. The resulting curve is similar to the exact solution **B**. The small insert shows the difference between the two methods. The error of the Monte Carlo approximation rises with the first unfolding events but stays well within a 2pN range. The probability function and the normalized histograms (bin size based on the Freedman-Diaconis rule [44]) show similarities in shape.

The comparison between Monte Carlo approximation based on the simulation of 200 titin strands and the exact solution reveals that mean forces of the Monte Carlo simulation and the exact solution correspond almost perfectly. In fact, the difference between the mean force of 200 titin strands and the exact solution stays within a range of 2pN for stretches from 1 to 2μm half sarcomere length. The comparison of the unfolding probabilities as a function of half sarcomere length and the corresponding histograms (bin size based on the Freedman-Diaconis rule [44]) reveal that the shape of the exact solution is preserved by Monte Carlo simulations (Fig 4).

**Fig 5:**
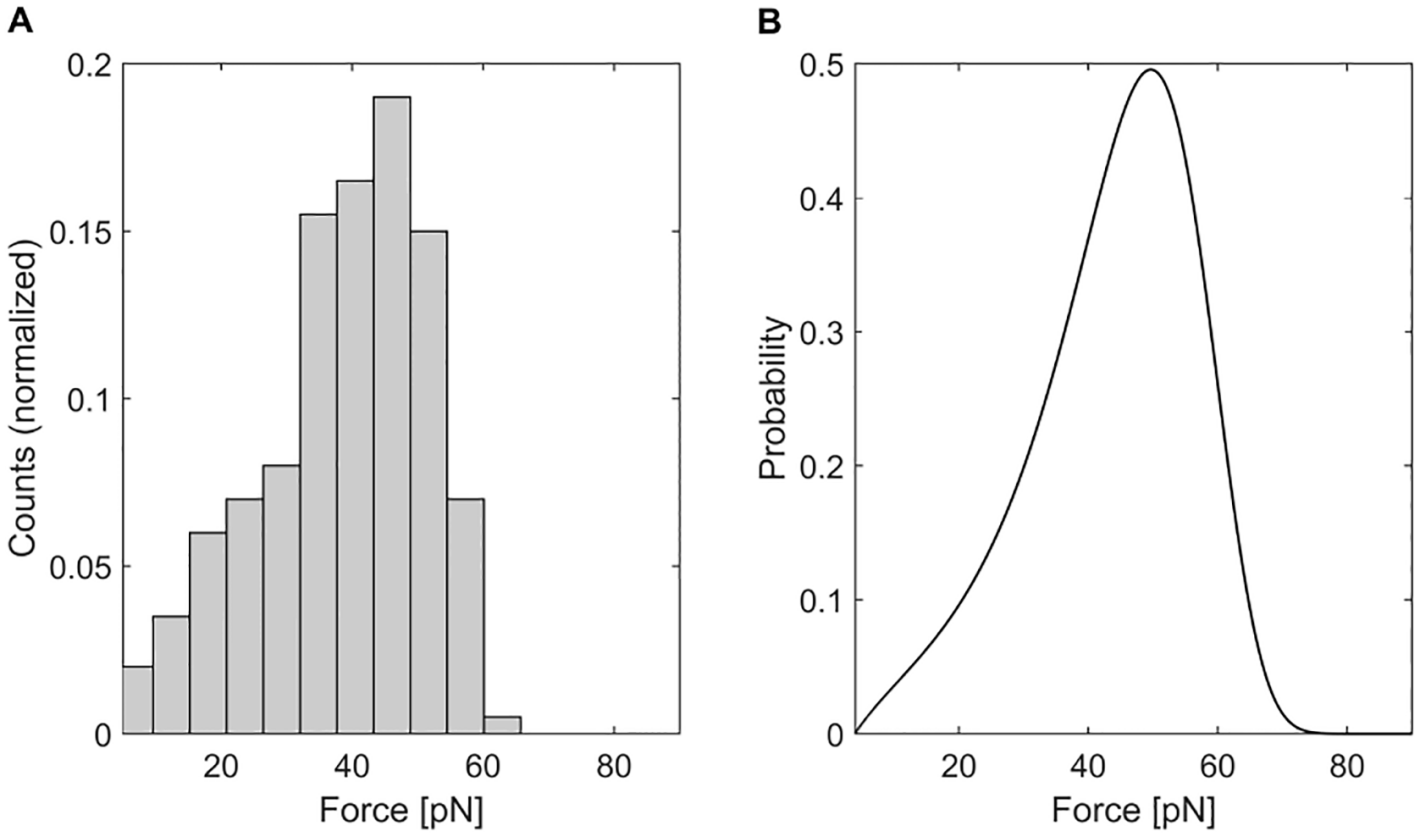
Forces at the first unfolding event. **A** Histogram of forces at the first unfolding event (bin size based on the Freedman-Diaconis rule [44]) of 200 Monte Carlo simulation reveal a deviation (in terms of most likely unfolding forces) from the corresponding exact probability **B**.

While the characteristics of the expected forces are comparable between Monte Carlo approximation and the exact solution other characteristics, like the most likely force at the first unfolding event, deviate (Fig 5) demonstrating that the simulation of more than 200 titin strands is necessary to capture the details of complex unfolding dynamics of all titin strands in a half sarcomere.

**Fig 6:**
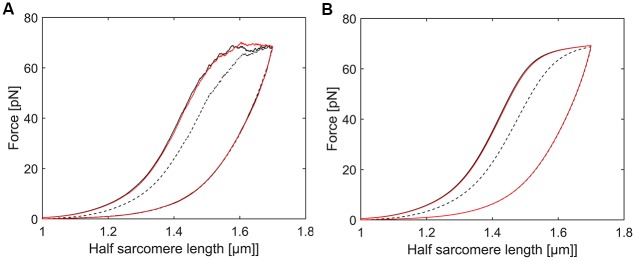
Repeated hysteresis loops. **A** Monte Carlo simulations and **B** exact solution of hysteresis loops based on one Ig cluster of the following setup: a half sarcomere was subject to two subsequent stretch-shortening cycles. After a resting period of 30s another stretch-shortening cycle was performed. We observed that while hysteresis was significantly reduced in the second cycle it fully recovered in the third cycle due to refolding of Ig domains in the resting period [5]. The effect of repeated stretch-shortening cycles on the unloading energy remains negligible.

Furthermore, the complex folding and refolding behaviour of Ig domains is captured with both methods. As an example, we simulated the following experiment which was based on a study on single titin molecules reported in [5] (Fig 6): A half sarcomere is stretched from 1μm to 1.7μm half sarcomere length and re-shortened from 1.7μm to 1μm in two subsequent cycles. After a rest period of 30 seconds a third stretch-relaxation cycle was performed. Hysteresis declined in the second compared to the first cycle, but was fully recovered in the third cycle following a 30s rest; in single titin molecule experiments, the hysteresis in the third cycle even exceeded the hysteresis observed in the first cycle. Its worth pointing out that, unlike single titin molecule experiments [5], we cannot predict higher forces in any cycle following the first one with our exact solution, as we assume that all Ig domains are folded at the start of our simulations (Fig 6).

**Fig 7:**
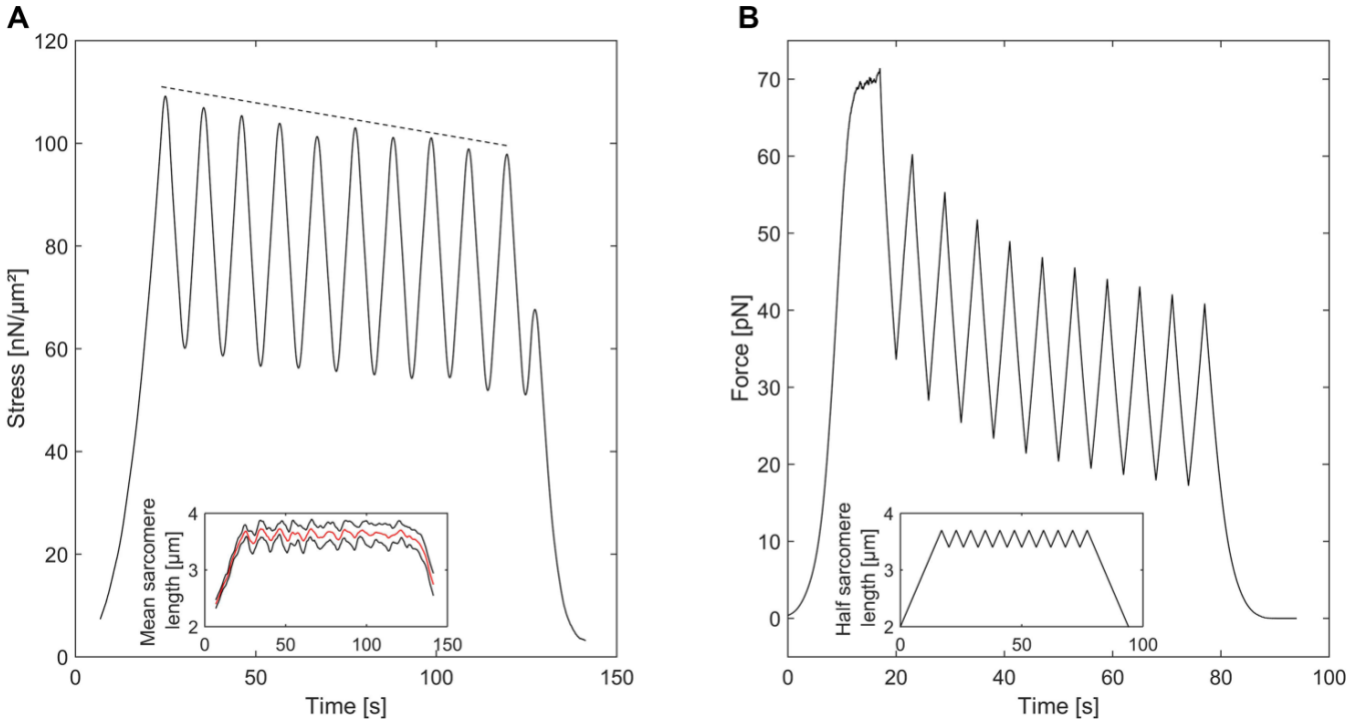
Hysteresis cycles of a single myofibril. **A** A single myofibril consisting of 7 sarcomeres was stretched to an averaged sarcomere length of 3.7μm and subsequently cycled by approximately ± 2.5μm. The small insert shows the corresponding average sarcomere length (red line) ± standard error (black lines). The peak forces declined from the first to the second last peak. **B** The average force of Monte Carlo simulations based on 200 titin strands and a simplified half sarcomere length behavior (small insert) show a decline of peak forces based on the folding / unfolding behavior of Ig domains.

Due to the architecture of a single myofibril, where a (possibly large) number of half sarcomeres are aligned in series, the comparison between simulations on the half sarcomere level and experimental results on the myofibril level have to be done carefully. However, since the main characteristics of the simulations should be preserved on the myofibrillar level, the comparison gives us the opportunity to compare model predictions to the passive behaviour of sarcomeres in their natural environment. As an example, we stretched five single myofibrils to an average sarcomere length of approximately 4μm and then immediately performed 10 stretch-shortening cycles of an amplitude of around 0.25μm. We can observe a typical decline in peak forces over the 10 stretch-shortening cycles [45]. The corresponding Monte Carlo simulations (based on 200 titin strands) show a comparable qualitative decline of peak forces; the results differ quantitatively as we do not take sarcomere non-uniformities into account. Moreover, we presumed a simplified length behavior to prevent artefacts (see Fig 7).

## Discussion

We were interested in an efficient algorithm to compute average passive forces in half sarcomeres. We analysed the model by seeking to recover primary characteristics of experiments of single titin molecules as well as single myofibrils rather than comparing absolute values. We neither performed parameter estimation, nor did we try to fit experimental data. However, in terms of parameter estimation, the proposed algorithm should prove a highly useful alternative to classic Monte Carlo simulations.

There are certain limitations to the proposed algorithm for determining the exact solution. When the number of Ig clusters is high, Monte Carlo simulations might be the faster option. On the other hand, the smaller the number of clusters, the faster is the calculation of the exact solution compared to the simulation of hundreds of titin strands. In the extreme case, when each Ig domain is attributed a significantly different mechanical stability state and allowed to refold, the exact solution is numerical expensive for long stretches. Therefore, Monte Carlo simulations are the preferred approach. On the other hand, if one is interested in the probability distributions of unfolding forces of the first unfolding events, the exact solution still provides an efficient framework. The same is true for parameter estimations.

**Fig 8:**
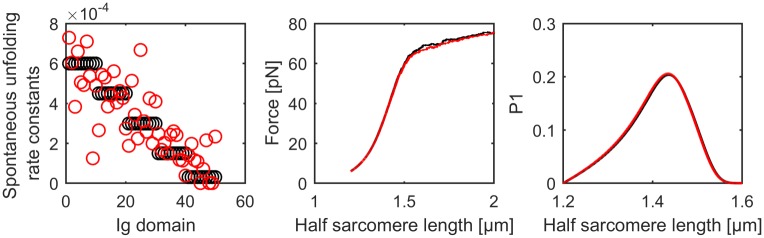
Comparison of different Ig clusters. The simulations are based on 50 Ig clusters (red) and 5 Ig clusters (black), respectively. The spontaneous unfolding rate constant (left panel) declines linearly but is perturbed by a Gaussian noise (SD = 0.2) in the case of 50 Ig clusters. The average force based on the Monte Carlo simulations of 50 titin strands (middle panel) as well as the probabilities of unfolding events as a function of half sarcomere length (right panel) are comparable between 50 Ig clusters (red) and 5 Ig clusters (black).

We introduce domain clusters in order to simplify the complexity of the folding / refolding process. While a limited number of clusters is favourable in terms of calculation time, it might be an over simplification. To study whether this simplification has a significant impact on the simulation outcome, we compare average forces and the probability of the first three unfolding events for two different cluster models. First, we look at 50 different clusters with linearly declining spontaneous unfolding rate constants and a random Gaussian perturbation and compare the results to simulations based on 5 different clusters with equidistant spontaneous unfolding rate constants. Since the overall shape of spontaneous unfolding rate constants are fairly similar for the two simulations, the corresponding average forces and unfolding forces are also remarkably similar (Fig 8).

However, clusters have to be chosen carefully. While the choice of the right clusters is of less importance when looking at qualitative behaviour of our system—we could perfectly explain hystereses of single titin molecules with one cluster—it is crucial in terms of parameter estimation or quantitative analysis. A perfect example would be the low force unfolding reported in a recent study by Martonfalvi et al [5]. The unfolding rates and first Ig clusters have to be chosen accordingly in order to cover the whole range of unfolding behavior that has been observed experimentally.

Finally, we would like to point out that the framework presented in this work is independent of the force model and the unfolding / folding rate functions used. Our approach is applicable whenever the average force of complex proteins is of interest.
